# Gestational diabetes and overt diabetes first diagnosed in pregnancy: characteristics, therapeutic approach and perinatal outcomes in a public healthcare referral center in Brazil

**DOI:** 10.20945/2359-3997000000310

**Published:** 2020-11-09

**Authors:** Yanara Sampaio, Lara Benigno Porto, Thais Cabral Gomes Lauand, Larissa Pereira Marcon, Hermelinda Cordeiro Pedrosa

**Affiliations:** 1 Unidade de Endocrinologia e Centro de Pesquisa Hospital Regional de Taguatinga Secretaria de Saúde do Distrito Federal Brasília DF Brasil Unidade de Endocrinologia e Centro de Pesquisa, Hospital Regional de Taguatinga, Secretaria de Saúde do Distrito Federal, Taguatinga, Brasília, DF, Brasil

**Keywords:** Gestational diabetes mellitus, overt diabetes, pregnancy, insulin, perinatal outcomes

## Abstract

**Objective:**

To describe the clinical characteristics, management, and fetal outcomes of patients diagnosed with gestational diabetes mellitus (GDM) or overt diabetes (OD) during pregnancy who followed up at a public healthcare referral center in Brazil.

**Materials and methods:**

A retrospective cohort study based on the medical records of women diagnosed with dysglycemia during pregnancy between January 2015 and July 2017 was conducted.

**Results:**

Out of 224 pregnant women evaluated, 70% were overweight/obese. GDM was observed in 78.6% of pregnant women, while 21.4% presented with OD. Approximately 59% of patients could be diagnosed with GDM or OD by fasting plasma glucose (FPG) alterations alone. Exclusive diet therapy was used in 50.9% of patients. The need for insulin therapy was higher in OD patients (60.4%) than in GDM patients (38.1%) (p = 0.006). Women who needed insulin (n = 96) had a mean initial dose of 0.33 IU/kg (±0.27) and a final value of 0.39 IU/kg (±0.34). The cesarean rate was 74.3%. The fetal outcomes evaluated were macrosomia (2.15%), large for gestational age (LGA) fetus (15.83%), intensive care unit (ICU) need (4.32%), Apgar score ≤7 (6.47%), hypoglycemia (14.39%) and jaundice (16.55%).

**Conclusions:**

Patients with GDM and OD presented with several similar clinical features. Approximately half of the patients presented with adequate glycemic control only with diet management. Patients with OD presented a higher need for insulin therapy. Although overweight and obesity were frequent within both groups, they could possibly explain many of our findings.

## INTRODUCTION

Elevated glucose levels during pregnancy are associated with a high risk of unfavourable obstetric and perinatal outcomes (1). The magnitude of hyperglycemia promotes different consequences, and the differential diagnosis between overt diabetes (OD) during pregnancy and gestational diabetes mellitus (GDM) itself should be performed because these are distinct clinical conditions (1,2).

In turn, hyperglycemia during pregnancy is a treatable condition, and adequate metabolic control may reduce the risk of complications (3,4). Dietary counselling associated with physical activity and glycemic monitoring are the first and fundamental aspects of the therapeutic approach (5,6). When nonpharmacological treatment is insufficient, as in up to 53% of the cases, although some studies indicate a prevalence of failure of approximately 20% to 30%, drug therapy is then recommended (6,7).

Brazilian data on the diagnosis and management of dysglycemia in pregnancy are scarce, especially those from the public health network. Thus, the present study aimed to describe real-life data concerning the main clinical characteristics of and the therapeutic approach and most relevant perinatal outcomes in patients with GDM and OD who were followed at a referral center of the public health network of the Federal District, Brazil.

## MATERIALS AND METHODS

A retrospective cohort study was carried out at the Specialized Outpatient Clinic of Gestational Endocrinopathies at the Regional Hospital of Taguatinga, Federal District. Medical data for patients who had a diagnosis of GDM or OD with a follow-up between January 2015 and July 2017 were obtained through a chart review.

For diagnosis, the criteria recommended by the Brazilian Society of Diabetes (8), which establish that patients presenting with the following alterations are diagnosed with GDM, were used: FPG ≥ 92 mg/dL and ≤ 126 mg/dL or an oral glucose tolerance test (OGTT) with 75 g of anhydrous glucose presenting one altered point (fasting ≥ 92 mg/dL, after 1 hour ≥ 180 mg/dL, and after 2 hours ≥ 153 mg/dL). On the other hand, patients with a diagnosis of OD were those with a fasting plasma glucose (FPG) ≥ 126 mg/dL and a 2-h plasma glucose on an OGTT ≥ 200 mg/dL. The goal of treatment adopted at our service is to maintain pre-prandial blood glucose lower than 95 mg/dL and postprandial blood glucose lower than 140 mg/dL after one hour using blood glucose self-monitoring (8).

Despite the orientation of blood glucose measurements one and two hours after 75-g anhydrous glucose stimulation at diagnosis, most of the laboratories of the Secretariat of Health of the Federal District use a protocol that includes measurements of FPG and 2-h plasma glucose on an OGTT only. For this reason, it was not possible to include blood glucose measurements after one hour.

Data were collected according to the chart number, date of birth, date of last menstruation, gestational age at diagnosis and at the first consultation with an endocrinologist, blood glucose values on diagnostic tests, body mass index (BMI) prior to gestation, weight gain, type of treatment, insulin dosage, birth data (gestational age, type of delivery and birth weight) and main fetal outcomes (hypoglycemia defined by glycemic levels lower than 50 mg/dL, need for intensive care unit (ICU), clinically diagnosed jaundice confirmed with laboratory tests and Apgar score ≤ 7).

We considered a fetal weight greater than 4 kg to indicate a macrosomic fetus, and a large for gestational age (LGA) fetus was indicated when the fetal weight was above 90th percentile according to gestational age. A local intrauterine growth pattern chart, which has been validated and is broadly used in the Federal District, was employed to determine the percentile of each newborn (9).

Descriptive statistics were used to present the clinical and demographic variables of the patients. Absolute and relative frequencies, means and standard deviation, and median and interquartile intervals were used as appropriate. Differences in the distribution of categorical variables were analysed with the chi-square test. The Shapiro-Wilk test was used to verify whether continuous variables were normally distributed. Parametric continuous variables were evaluated with a t test, and nonparametric variables were evaluated with the Mann-Whitney test. The Spearman correlation test was used to evaluate correlations between the variables. Poisson regression analysis was employed to calculate the risk ratio of factors associated with insulin treatment. The level of significance was set at a p-value <0.05. Analyses were performed using the Statistical Package for Social Sciences (SPSS), version 22.0.

This work was approved by the Local Research Ethics Committee under the Certificate of Presentation for Ethical Appreciation (CAAE) number 75695917.7.0000.5553/Approval Number 2.283.783.

## RESULTS

A total of 234 patients were diagnosed with GDM and OD between January 2015 and July 2017. Ten were excluded due to insufficient data. Therefore, 224 patients were evaluated.

The general characteristics of the sample are shown in Table 1. In regards to their pre-gestational BMI, 77 patients (34.4%) were obese, 77 (34.4%) were overweight, 58 (25.9%) had a normal BMI, 1 (0.4%) was underweight, and 11 (4.9%) did not have information on their pre-pregnancy weight.

**Table 1:** Comparison of the characteristics between GDM and OD patients

Characteristics	Overall (n = 224)	GDM (n = 176)	OD (n = 48)	P-value*
Age (years)^a^	33.00 (±7.00)	33.00 (±7.00)	31.00 (±10.00)	0.267
GA at diagnosis (weeks)^a^	26.00 (±9.00)	26.00 (±9.00)	26.07 (±8.46)	0.765
GA at first appointment (weeks)^a^	32.57 (±6.36)	32.86 (±6.57)	32.21 (±6.25)	0.243
Pre-gestational BMI (kg/m²)^a^	28.00 (±7.54)	27.97 (±7.94)	28.40 (±6.35)	0.353
Gestational weight gain (kg)^a^	8.15 (±7.40)	8.00 (±6.80)	9.30 (±8.70)	0.445
Need for insulin therapy^b^	96 (42.8)	67 (38.1)	29 (60.4)	0.006**
Initial dose of insulin IU/kg^a^	0.23 (±0.39)	0.17 (±0.25)	0.53 (±0.56)	<0.001**
Final dose of insulin IU/kg^a^	0.28 (±0.44)	0.19 (±0.29)	0.55 (±0.53)	0.002**

GDM: gestational diabetes mellitus; OD: overt diabetes; GA: gestational age; BMI: body mass index; FGP: fasting plasma glucose; OGTT: oral glucose tolerance test.

a Values expressed as median (± interquartile range).

b Values expressed as proportion n (%).

* P-values were based on Mann-Whitney test (skewed continuous variables) or chi-square test (categorical variables).

** P-value < 0.05. Number of patients who needed insulin therapy and doses of insulin were significantly different through the comparison between GDM and OD groups.

A hundred and seventy six women (78.6%) met the criteria for GDM, while 21.4% presented with OD. The main characteristics of GDM and OD groups are presented in Table 1. Patients with OD presented a higher need for insulin therapy and higher insulin doses. Age, gestational age (GA) at diagnosis, GA at first appointment, pre-conception BMI and weight gain were not significantly different between the groups.

Regarding glucose at diagnosis, approximately 67 patients (30%) showed high FPG (measured alone or during the OGTT), 92 (41%) showed high blood glucose after 2 hours on the OGTT, and 65 (29%) presented changes in both. There was a delay of approximately 7 weeks from when the exams were performed to the first medical evaluation at our center.

Exclusive diet therapy was used in 114 patients (50.9%), diet plus insulin therapy was used in 96 (42.9%), and diet plus oral antidiabetic therapy was used in 14 (6.2%). Among those on antidiabetic drugs, 12 used metformin, 1 used glibenclamide, and 1 used glibenclamide associated with metformin. Among the 96 patients who received insulin, a total of 7 (7.3%) were on metformin as well.

When insulin-treated (n = 96) vs. non-insulin-treated (n = 128) patients were compared (Table 2), insulin-treated patients showed higher FPG, 2-h plasma glucose on an OGTT, and weight gain during pregnancy and a higher prevalence of OD.

**Table 2:** Comparison between insulin-treated and non-insulin-treated patients

	Insulin-treated group (n = 96)	Non-insulin-treated group (n = 128)	P-value*
Age (years)^a^	34.00 (±9.00)	32.00 (±7.00)	0.407
GA at diagnosis (weeks)^a^	26.00 (±8.86)	26.00 (±8.75)	0.828
GA at 1st appointment (weeks)^a^	32.43 (±6.50)	32.71 (±6.25)	0.478
Pre-gestational BMI (kg/m²)^a^	28.32 (±6.40)	27.57 (±8.07)	0.201
Gestational weight gain (kg)^a^	9.35 (±6.90)	6.90 (±7.00)	0.038**
FPG at diagnosis (mg/dL)^a^	98.00 (±26.00)	93.00 (±18.00)	0.004**
2-h plasma glucose on OGTT (mg/dL)^a^	167.50 (±38.00)	165.00 (±25.00)	0.033**
Overt diabetes frequency^b^	29 (30.2)	19 (14.8)	0.006**

FGP: fasting plasma glucose; OGTT: oral glucose tolerance test; GA: gestational age; BMI: body mass index.

a Values expressed as median (± interquartile range).

b Values expressed as proportion n (%).

* P-values were based on Mann-Whitney test (skewed continuous variables) or chi-square test (categorical variables).

** p < 0.05.

The regression model (data not shown) to evaluate risk factors for insulin treatment did not show any association with the type of diabetes (GDM or OD), age, GA at diagnosis, GA at the first consultation, BMI preconception, weight gain, FPG or 2-hour plasma glucose on an OGTT. This means that no explanatory variable was significant in the Poisson regression model using insulin as a response.

Women who needed insulin (n = 96) had a mean initial dose of 0.33 IU/kg (±0.27) and a final value of 0.39 IU/kg (±0.34) (data for GDM and OD groups are displayed in Table 1). There was a correlation between FPG at diagnosis and the initial dose of insulin (r = 0.511; p < 0.001) and between FPG at diagnosis and the final dose of insulin (r = 0.526, p < 0.001). Among patients with OD (n = 48), 19 (39.6%) obtained glucose control without insulin, although in 3 cases, metformin was associated with the diet.

After assessing medical records, it was possible to obtain data on gestational age and type of delivery in 171 women (76.3%). The mean gestational age at delivery was 38.45 ± 1.86 weeks, and cesarean sections occurred in 127 cases (74.3%). The insulin-treated patients showed a lower gestational age at delivery (median 38.00 ± 1.11 weeks) than patients who were treated by diet (median 39.00 ± 1.89) (p < 0.001). A higher prevalence of cesarean section was also observed (p = 0.003) when comparing insulin-treated [available data for 71 women, cesarean section in 61 (85.9%)] vs. non-insulin-treated patients [available data for 100 women, cesarean section in 66 (66%)]. Regarding postpartum re-evaluations, only 71 patients (31.7%) returned for outpatient follow-up.

Medical records of the fetal outcomes were obtained for 139 patients (62.1%). The main outcomes are displayed in Table 3.

**Table 3:** Main perinatal outcomes (n = 139)

Perinatal Outcomes	Overall (n = 139)	GDM group (n = 105)	OD group (n = 34)
Birth Weight (g)^a^*	3,266.24 ± 468.61	3,279.21 ± 411.21	3,222.66 ± 610.66
LGA (n)^b^	22 (15.83)	14 (13.33)	8 (23.53)
Macrosomia (n)^b^	3 (2.15)	1 (0.95)	2 (5.88)
Need for ICU (n)^b^	6 (4.32)	2 (1.90)	4 (11.76)
Apgar score ≤ 7 (n)^b^	9 (6.47)	6 (5.71)	3 (8.82)
Hypoglycemia (n)^b^	20 (14.39)	12 (11.43)	8 (23.53)
Clinical jaundice (n)^b^	23 (16.55)	17 (16.19)	6 (17.64)

OD: overt diabetes; GDM: gestational diabetes mellitus; LGA: large for gestational age; ICU: intensive care unit.

a Values expressed as mean (± standard deviation).

b Values expressed as proportion n (%).

* P-value = 0.552 (Result obtained from Student's t-test) when comparing GDM vs. OD. Differences in the other outcomes were not evaluated because of the small number of patients with complications in each group.

## DISCUSSION

The recent change in the diagnostic criteria raised the prevalence of dysglycemia in pregnancy (10,11), but the concomitant increase in obesity and metabolic syndrome leads to implications in this scenario as well (7). Overweight in pregnant women with diabetes is associated with deleterious maternal-fetal outcomes (7,12,13). In the present study, the pre-gestational BMIs indicated that 70% had overweight or obesity.

As expected, there was a higher prevalence of GDM than OD at diagnosis (Table 1), occurring in approximately 79% of the cases. Similar findings have been shown in other series (14,15). There was an important delay between laboratory diagnosis and specialized care, which reflects the need to improve assistance.

In 2016, the Pan American Health Organization, the Ministry of Health and the Brazilian Federation of Gynecology and Obstetrics together with the SBD announced new recommendations for the screening and diagnosis of GDM and OD, considering the reality of public health and the latest World Health Organization criteria, and all were within the recommendations of the International Association of Diabetes and Pregnancy Study Groups (IADPSG) (2). This new guideline proposes a flowchart for situations in which technical or financial availability are limited, by recommending the exclusive use of FPG as a diagnostic tool for GDM and OD during pregnancy.

The use of FPG alone for the diagnosis of gestational diabetes was reviewed by Dickinson in 2019 (16). The difference in test performance depends on the population studied. Better performance was seen in Caucasians than in South Asians. When applied to the HAPO study, FPG would diagnose only approximately 55% of GDM cases. In our sample, only 59% of pregnant women could be diagnosed by FPG, which does not reach the expectation of the guideline, which estimates coverage of 86% (2). The Brazilian Study of Gestational Diabetes retrospectively analysed FPG as a tool to avoid the OGTT using a cohort from 1991 to 1995 and found a sensitivity of 86%. However, their population was younger (mean maternal age 27.8 years) and lighter (mean BMI at enrolment 26 kg/m^2^) (17) than ours (median maternal age 33 years and pre-conception BMI 28 kg/m^2^). This could reflect 20 years of the obesity epidemic and changes in aspects of fertility. Perhaps this point should be better evaluated in further studies, especially when considering its impact on public health.

Pharmacological treatment was necessary for 49% of the patients. This finding is consistent with other studies in which failure rates with nonpharmacological treatment may reach 53% (6,7). An interesting point is that 39.6% (n = 19) of the patients diagnosed with OD (n = 48) achieved the therapeutic goal without the use of insulin.

There are several studies examining oral antidiabetic drugs during pregnancy, mainly metformin and glibenclamide (18-20). Evidence favouring metformin instead of glibenclamide has been reported (19,21), although it was not confirmed (20). Metformin is a cheap and easy-to-use drug that helps up to 66% of women with GDM, but since its long-term safety is still questioned, the first choice is still insulin (22).

There are studies demonstrating that some clinical and laboratory parameters may predict the need for insulin therapy (6,7). Although we found that weight gain, FPG, 2-h plasma glucose on the OGTT, and the diagnosis of overt diabetes presented a statistically significant association with insulinization individually (Table 2), which is consistent with previous studies (6,7), the regression model did not show multiple associations with these characteristics.

For patients who needed insulin, the final dose was approximately 0.4 IU/kg. This result suggests that low insulin doses may be sufficient in many cases. This finding can be related to the fact that the new diagnosis criteria proposed by IADPSG (1) include many patients with milder glucose elevations.

An alarming finding was the high prevalence of cesarean deliveries (74.3%). Brazil is second in the world in terms of the number of cesarean sections (23). In studies with patients with GDM or OD, the incidence of cesarean deliveries ranges from 23.7% to 51.4% (4,5,14). The presence of insulinization seemed to promote a greater indication for cesarean section in the present study.

Also noteworthy was the low postpartum outpatient return rate, which was approximately one third of the women, indicating that it is essential to implement strategies to improve follow-up, considering the importance of ensuring continuality of care in OD patients and preventing type 2 diabetes in GDM cases (24-26).

Regarding the main fetal outcomes, there was a low prevalence of macrosomia (2.15%), and the rate of LGA newborns (15.83%) was comparable to that from other clinical studies (4,13,27). On the other hand, the need for ICUs (4.32%) was lower than that found in the literature, which varies between 9.6 and 35% (14,28).

The present study has some limitations, such as its retrospective nature, the small number of cases evaluated and the lack of the 1-hour values for OGTTs in most of our data. Despite the absence of novelty, our study addresses important issues because it reflects the real-life daily routine at a specialized center of the public health network in Brazil after the adoption of IADPSG (1) criteria for GDM diagnosis. This is particularly important when considering that daily clinical practice should be adapted to the available resources in each scenario according to current guidelines (2,22).

In conclusion, patients with GDM and OD presented with several similar clinical features. Approximately half of the patients presented with adequate glycemic control only with diet management. Patients with OD presented a higher need for insulin therapy and insulin-treated patients presented with a higher rate of cesarean section. Although overweight and obesity were frequent within both groups, they could possibly explain many of our findings.
